# The structure of mania: An overview of factorial analysis studies

**DOI:** 10.1192/j.eurpsy.2020.18

**Published:** 2020-02-10

**Authors:** Diego J. Martino, Marina P. Valerio, Gordon Parker

**Affiliations:** 1 Institute of Cognitive and Translational Neuroscience (INCyT), INECO Foundation, Favaloro University, Ciudad Autónoma de Buenos Aires, Argentina; 2 National Council of Scientific and Technical Research (CONICET), Buenos Aires, Argentina; 3 Psychiatric Emergencies Hospital Torcuato de Alvear, Buenos Aires, Argentina; 4 School of Psychiatry, University of New South Wales, Sydney, New South Wales, Australia

**Keywords:** depression, diagnostic criteria, mania, mixed states, nosology

## Abstract

**Background.:**

Operational definitions of mania are based on expert consensus rather than empirical data. The aim of this study is to identify the key domains of mania, as well as the relevance of the different signs and symptoms of this clinical construct.

**Methods.:**

A review of latent factor models studies in manic patients was performed. Before extraction, a harmonization of signs and symptoms of mania and depression was performed in order to reduce the variability between individual studies.

**Results.:**

We identified 12 studies fulfilling the inclusion criteria and comprising 3039 subjects. Hyperactivity was the clinical item that most likely appeared in the first factor, usually covariating with other core features of mania, such as increased speech, thought disorder, and elevated mood. Depressive–anxious features and irritability–aggressive behavior constituted two other salient dimensions of mania. Altered sleep was frequently an isolated factor, while psychosis appeared related to grandiosity, lack of insight and poor judgment.

**Conclusions.:**

Our results confirm the multidimensional nature of mania. Hyperactivity, increased speech, and thought disorder appear as core features of the clinical construct. The mood experience could be heterogeneous, depending on the co-occurrence of euphoric (elevated mood) and dysphoric (irritability and depressive mood) emotions of varying intensity. Results are also discussed regarding their relationship with other constitutive elements of bipolar disorder, such as mixed and depressive states.

## Introduction

In his original description of manic-depressive insanity, Kraepelin [1] proposed that the structure of mania was based on three fundamental clinical features: euphoria, pressured speech, and hyperactivity. This triad remained among the signs and symptoms that expert textbook authors primarily considered them for the diagnosis of mania in western psychiatry until 1960 [2]. Later, when formalized diagnostic criteria for mania were generated, some of them retained the focus on the presence of all three clinical features [3] or, alternatively, on only two of them [4]. It is important to highlight that, throughout this period, no primacy was given to any sign or symptom over another, which made it possible to diagnose mania without any elevation of mood [4].

This historical perspective changed with the publication of the Feighner criteria [5], the Research Diagnostic Criteria [6], DSM-III (APA, 1980) and successive DSM editions, all of which prioritized an elevated, expansive, or irritable mood (Criterion A) over other manic symptoms and signs (Criterion B). These operational definitions were based on expert consensus rather than empirical data [7], and subsequent studies showed the relevance of activation as a clinical feature of mania as reviewed by Scott et al. [8]. The prominence of such mood abnormalities was partially reversed in the DSM-5 (2013), with bipolar disorder (BD) and related conditions being removed from the mood disorders chapter, and persistent increased activity or energy was included as part of Criterion A for the diagnosis of mania. However, the empirical support for the other criteria included in the current definition of mania is limited.

One empirical approach for studying and clarifying key clinical features of mania is to undertake factor analytic studies. Factor analysis, reduces multiple correlated variables (i.e., different clinical items) to fewer latent dimensions or factors that define the key constructs. Thus, factor analysis has the capacity to clarify how manic features covary, as well as the relative relevance of each sign or symptom (by their loadings) to defining identified dimensions. Although factor analytic studies of manic features have shown the multidimensional nature of the syndrome, findings have been inconsistent [9–13]. The lack of consensus in defining the factors underlying mania could reflect several methodological issues, such as some studies including non-manic subjects; differences in age of the patients (i.e., children–youth vs. adults); differing sets of manic symptoms and signs (with or without sets of depressive or non-affective features); and reliance on self-report data that can be less reliable and valid than interview methods (particularly when manic patients may lack insight). In order to overcome some of these limitations, we conducted a review of factor analytic studies to determine if a dominant or relatively consistent model could be identified for defining the key constructs of mania, as well as the relative relevance of individual clinical items to each construct.

## Methods

### Search strategy and study selection criteria

A comprehensive search of the literature was undertaken by accessing PubMed and PsycINFO databases on August 10, 2019 using combinations of the following search terms: “bipolar” or “mania” or “manic” and “factorial” or “principal component”, restricted to English studies in human individuals. Two reviewers (D.J.M. and M.P.V.) independently conducted the title/abstract screen and the subsequent full text assessment. Areas of disagreement were resolved by discussion until consensus was reached.

Studies were included in the present review if they met the following criteria: (a) were published in a peer-reviewed English language journal; (b) assessed a clinical sample of subjects aged 18 years or older and who were experiencing a manic episode diagnosed by operationalized criteria; (c) employed interview methods (i.e., health professional-rated scales) to assess clinical variables (studies using self-reported scales were excluded); and d) used latent factor models, such as analysis of principal components or exploratory factorial analysis to define syndrome dimensions. For studies with overlapping content based on the same patient sample, only the data from the study with the largest sample were considered. Since our review focuses on the structure of mania, we excluded those studies that included combined samples of patients with manic and mixed episodes, and that did not report the results of the factor analysis separately for these subsets of patients [11,14–17].

#### Data extraction

Data extraction was performed in duplicate by two reviewers (D.J.M. and M.P.V.). The main information extracted and reported from each study were: the number of factors, the composition and amount of variance explained by each factor, and the amount of variance explained overall. Before extraction, the variability of data from different studies were reduced to a subset of more homogeneous manic symptoms and signs. This data harmonization was carried out by adapting the clinical items assessed in each study to a list of 22 historical clinical features of mania reported in a recent review [2]. This baseline list was supplemented with four additional manic features—increased sociability, psychomotor agitation, disorientation, and impaired functioning—as well as 12 depressive and anxious signs and symptoms, extracted where relevant from our reviewed studies. Five clinical variables were listed in both mania and depression scales: distractibility, altered sleep, appetite, psychomotor agitation, and impaired functioning. The final list of 37 clinical variables included in our review is shown in Table 1.

**Table 1.** List of synonymous signs and symptoms used for the harmonization of clinical variables extracted from reviewed studies

Signs/symptoms	Synonymy	Mania	Mixed
Elevated mood	Euphoric mood/sense of humor/looks happy and cheerful/euphoria/excitement/elated mood	12	7
Hyperactivity	Increased motor activity–energy/moves from one place to another/is active/absence of fatigue	12	7
Increased speech	Increased rate and quantity of speech/is talking/pressured speech/accelerated speech	12	7
Irritability	Is angry/is argumentative/makes threats/anger/impatience	12	7
Grandiosity	Has grandiose ideas/inflated self-esteem/exaggerated self-esteem/confident/optimistic	9	4
Poor judgments	Poor judgments in new activities/activities with high potential for painful consequences/makes unrealistic plans/bizarre behavior	4	1
Psychosis	Delusions/paranoia/hallucinations/unusual thought content/is suspicious/distrustfulness/item 8 (content) of the Young Mania Rating Scale	11	7
Distractibility	Is distractible/concentration difficult/inability to think	5	4
Thought disorder	Disorganization of speech/flight of ideas or racing thoughts/jumps from one subject to another/jumps subject/flight of thought/altered thinking and speech/conceptual disorganization	12	7
Altered sleep	Initial insomnia/middle insomnia/terminal insomnia/general insomnia/decreased need for sleep/decreased sleep	12	7
Change in moral standards		0	0
Feelings of well-being	Increased drive	2	0
Increased sexuality	Is sexually preoccupied/talks about sex/sexual interest	9	4
Appetite	Poor appetite/increased appetite	1	1
Hypergraphia		0	0
Weight changes	Weight loss, weight increased	0	0
Impulsivity	Has diminished impulse control	2	1
Appearance	Dresses inappropriately/is careless about dress and grooming/self-neglect	7	2
Mood lability	Is unstable	4	1
Lack of insight	Poorer insight	7	6
Disruptive–aggressive behavior	Is combative or destructive/aggression/hostility/uncooperativeness	10	5
Increased sociality	Demands contact with others/seeks out others/increased contact	6	3
Psychomotor agitation	Agitation/mannerisms and posturing/motor restlessness/inner tension	3	2
Disorientation		1	0
Impaired functioning	Reduced work	2	0
Depressed mood	Looks depressed/dysphoria/sad appearance/crying/reported and apparent sadness	9	7
Depressed feelings	Verbalizes depressive feelings/guilt/self-reproach/negative self-evaluation/discouragement/helpless–hopeless/feeling of impoverishment/rumination/inadequacy	7	6
Anxiety	Anxiety/worry/phobias/somatic anxiety/psychic anxiety/panic/obsessions/somatic concerns	4	5
Suicide	Suicidal tendencies	5	6
Social withdrawal		0	2
Fatigue	Tiredness and pains/loss of vitality	4	3
Anhedonia–loss of interest		1	1
Psychomotor retardation	Retarded movement and speech/motor retardation	1	2
Indecisiveness		0	1
Mood non-reactivity	Retardation (emotional)/apathy/blunted affect/emotional withdrawal/inability to feel/apathy	3	2
Memory impairment		0	1
Diurnal variation	Mood worse in morning/mood worse in afternoon	1	0

The mania and mixed columns indicate the number of studies in which the variable was analyzed.

Only items with absolute value loadings >0.4 were accepted in our analyses. When an item had a loading >0.4 in more than one factor in a given study, it was included in the factor, in which it had a higher absolute value. Apart from clinical variables, other extracted information from each individual study were: characteristics of study population (number of subjects, age, and clinical setting), criteria for the diagnosis of manic episode, mania items assessed and scale used for their assessment, type of latent factor model, and type of rotation. Quality of the study was assessed by means of the STROBE Statement Checklist [18].

## Results

After the exclusion of duplicated retrieves, we obtained 280 articles, from which 261 were excluded based on the titles and/or abstracts. The remaining 19 articles had their full text assessed to confirm or exclude with 8 studies meeting inclusion criteria. Finally, the reference lists of retrieved reports and previous reviews on the topic were hand-searched for further relevant investigations, which include of four additional studies to the review, obtaining a final number of 12 studies and comprising 3039 manic patients. Only 6 of our 37 clinical variables were included in all studies: elevated mood, hyperactivity, increased speech, irritability, thought disorder, and altered sleep. Three historical features of mania in Kendler’s review [2] were not included in any study: change in moral standards, hypergraphia, and weight changes. The main characteristics and the factors found in each study are shown in Table 2.

**Table 2.** Summary of studies included in the review

Study	*n*/setting/diagnosis	Items/no. of factors	Rotation	Factor 1/variance	Factor 2/variance	Factor 3/variance	Factor 4/variance	Factor 5/variance	Factor 6/variance	Factor 7/variance	Total variance
Manic studies											
[19]	81/int/[4]	26 items Manic-State Raring Scale/3	Varimax	Hyperactivity, impulsivity, increased sociality, thought disorder, increased speech, appearance/NS	Irritability, elevated mood, depressive feeling, depressed mood/NS	Grandiosity, psychosis, feelings of well-being, poorer judgments, disruptive-aggressive behavior/NS					
[10]	124/int/DSM-IIIR	22 items Bech-Rafaelsen Mania and Melancholia/5	Varimax	Hyperactivity, increased speech, thought disorder, increased sociality, increased sexuality, elevated mood, grandiosity/29.4%	Depressive feelings, depressed mood, fatigue, anxiety, suicide, impaired functioning/18.9%	Psychomotor retardation, non-reactivity mood/7.5%	Disruptive–aggressive behavior/6.0%	Altered sleep/4.9%			66.7%
[20]	100/out /ICD-10	20 items Scale for Manic States/3	Varimax	Hyperactivity, increased speech, thought disorder, increased sexuality, increased sociality/18.3%	Psychosis, grandiosity, lack of insight/14.0%	Irritability, disruptive–aggressive behavior, anxiety, elevated mood/13.8%					46.1%
[13]	104/int/DSM-IV	26 items Manic-State Raring Scale/7	Varimax	Increased sociality, hyperactivity, mood lability, thought disorder, increased speech/NS	Irritability, disruptive–aggressive behavior/NS	Appearance, distractibility, poor judgments/NS	Grandiosity, psychosis/NS	Elevated mood, Feelings of well-being/NS	Depressive mood, depressive feelings, impulsivity/NS	Increased sexuality/NS	
[9]	363/int /DSM-IV	12 items Young Mania Rating Scale and total score from Hamilton Depression Rating Scale score/5	Varimax	Hyperactivity, elevated mood, increased speech/24%	Psychosis, poor judgments, lack of insight/18%	Depressive mood (HDRS score), irritability/11%	Thought disorder/11%	Altered sleep/10%			73%
[21]	88/int/ICD-10	24 items Brief Psychiatric Rating Scale/4	Varimax	Hyperactivity, elevated mood, distractibility, grandiosity, poor judgments/13.74%	Mood non-reactivity, appearance, thought disorder, disorientation/13.16%	Psychosis/8.72%	Disruptive–aggressive behavior, psychomotor agitation/6.78				51%
[22]	225/out/ICD-10	20 items Scale for Manic State/6	Oblimin	Psychosis, grandiosity/17.01%	Irritability, disruptive–aggressive behavior, lack of insight, elevated mood/13.23%	Depressive mood, mood lability, anxiety/9.29%	Thought disorder, increased speech/7.63%	Increased sexuality, increased sociality/6.43%	Hyperactivity, altered sleep/5.70%		59.29%
[23]	131/int/ICD-10	11 items Young Mania Rating Scale/3	Promax	Irritability, hyperactivity, disruptive-aggressive behavior/NS	Thought disorder, elevated mood, increased sexuality, lack of insight/NS	Psychosis, appearance, altered sleep, increased speech/NS					
[12]	1535/NS/DSM-IV	21 items Young Mania Rating Scale and Montogomey-Asberg Depression Rating Scale/5	Varimax	Depressive mood, mood non-reactivity, depressive feelings, fatigue, suicide/19.0%	Increased speech, thought disorder, elevated mood, hyperactivity, psychosis/12.8%	Altered sleep, distractibility/7.8%	Lack of insight, appearance, increased sexuality/6.6%	Irritability, disruptive–aggressive behavior/5.7%			51.9%
[24]	117/int/DSM-IV, ICD-10	36 items Schedule for Affective Disorders and Schizophrenia/6	Promax	Depressive feelings, depressive mood, anxiety, fatigue, appetite, anhedonia-loss of interest/17.01%	Suicide, mood variation, impaired functioning/12.38%	Altered sleep/10.35%	Hyperactivity, elevated mood, grandiosity, Irritability/7.67%	Poor judgments, psychosis/6.34%	Anxiety, psychomotor agitation/5.55%		59.3%
[25]	96/int/DSM-IV	19 items Young Mania Rating Scale, Montogomey-Asberg Depression Rating Scale, and Scale for the Assessment of Positive Symptoms/3	Varimax	Hyperactivity, increased speech, thought disorder, disruptive-aggressive behavior, elevated mood, irritability, appearance, altered sleep, increased sexuality/NS	Depressive mood, suicide, depressive feelings, psychomotor agitation, fatigue/NS	Psychosis, lack of insight/NS					
[26]	75/int/DSM-IV	20 items Scale for Manic State/4	Varimax	Thought disorder, increased speech, elevated mood, hyperactivity, grandiosity, lack of insight, appearance/31.16%	Suicide, Depressive feelings, Depressive mood, Mood lability, Anxiety / 20.77%	Irritability, disruptive–aggressive behavior, psychosis/11.9	Increased sociality, increased sexuality/5.96				69.8%

The clinical features within each factor in the different studies were extracted in decreasing order of loadings (higher loading first).

Abbreviations: Int, intpatients; NS, not specified; Out, outpatients.

Hyperactivity emerged in the first factor in nine of the studies reviewed, and was also the variable with the highest loading in six. Increased speech and thought disorder frequently covaried with hyperactivity in the first factor, and with loadings greater than any mood abnormalities.

Elevated mood was the first factor in only five studies, whereas irritability emerged as the first factor in three studies. Elevated mood appeared more frequently in the factor that included hyperactivity, increased speech, and thought disorder. Irritability appeared associated with both elevated mood in some studies as well as in a separate domain linked to disruptive-aggressive behavior in others.

Anxious and depressive symptoms tended to covary, comprising the first factor in two studies and the second in three studies. Altered sleep was frequently an isolated factor, while psychosis appeared related to grandiosity, lack of insight, and poor judgment. Increased sociability and increased sexuality frequently covaried, in some studies, being associated with core features of mania and in others, emerging as a separate factor.

**Figure 1.**
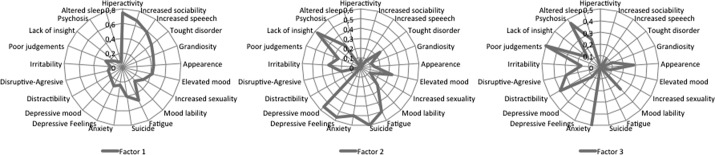
Spider diagram of the factor structure of mania (the unit of measure is the probability of the variable being included in factors 1, 2, and 3).

For those clinical variables evaluated in more than a third of the studies included in our review, the probability of being identified in the first three factors is shown in Figure 1.

Overall, quality of included studies was good, except for the lack of assessment of potential sources of bias and the lack of description of any missing data.

## Discussion

Compared to other disorders, such as schizophrenia or unipolar depression, there is a relative paucity of factor analytic studies of manic symptoms. In addition, despite our objective of harmonizing data from different published studies, there were a number of methodological issues that should be taken into account before interpreting our findings. First, a major limitation inherent to all studies that have explored the factorial structure of mania in naturalistic samples is that medication and different stages of recovery from the episode can confound symptom assessment. Similarly, due to the characteristics of the included individual studies, it was not possible to explore the influence of comorbidity (e.g., anxiety disorders) or age at illness onset on the reported factors. Second, the variables assessed in these studies should provide representative measures of each expected factor as the omission of key variables will seriously alter or obscure the factor solution [27]. In this sense, it should be noted that several clinical features that are seemingly characteristic of mania, such as distractibility, impulsivity, mood lability, or poor judgment were included in only a minority of studies. Likewise, depressive features were evaluated via an extensive set of symptoms and signs in some studies, while in others they were only evaluated as a global depression score or were omitted. In addition, the tools used in all these studies rely on a categorical approach and could miss possible relevant dimensions (e.g., sensory perception). Third, although most of the studies used the varimax rotation method, others used an extraction method with oblique rotation arguing that the factors tend to correlate with each other. Finally, in some studies, the number of patients was below the usual recommendation [28] for the generation of stable factors (i.e., being 10 times greater than the number of clinical items assessed). Reflecting these caveats, our results should be considered as indicative rather than definitive in seeking to determine the structure of mania.

### The structure of mania

A factor found with some consistency across the studies was one containing suggested core features of mania (i.e., items such as hyperactivity, increased speech, thought disorder, and elevated mood). In some studies, increased sociability and sexuality were associated with such core features of mania, whereas in others they comprised a separate factor. Hyperactivity was the feature most likely to be identified in the first factor of the studies, and had the highest loading in most. This finding is consistent with a recent review detailing the relevance of activation in BD [8], and with increased activity or energy being added to Criterion A for the diagnosis of mania in DSM-5 (2013). Likewise, both increased speech and thought disorder appeared more frequently and with greater loadings than elevated mood in the first factor of our reviewed studies. This finding is in agreement with a set of diagnostic criteria suggested by Taylor and colleagues [3], and which positioned elevated mood, hyperactivity, and rapid or pressured speech as having equivalent salience for the diagnosis of mania, as against successive iterations of the DSM manuals, in which increased speech and thought disorder were relegated to the symptoms of Criterion B. Importantly, our results do not empirically support the primacy given to elevated or expansive mood for the diagnosis of mania in both the Feighner criteria and DSM editions from DSM-III to DSM-5.

Other mood abnormalities generated different factors. Irritability was frequently associated with disruptive–aggressive behavior in a separate factor, whereas depressive–anxious features emerged as another salient dimension. These results are consistent with the review by Goodwin and Jamison [29] that found a high prevalence of irritability, mood lability, and depression during manic episodes. Different empirical approaches also converge in showing the coexistence of euphoric and dysphoric emotions during manic episodes. Based on a small factor analytic study, Murphy et al. [30] concluded that mania does not seem well characterized by elation, but more by a state of overall activation with enhanced affective expression together with lability of affect. Similarly, based on a principal component analysis combining clinical assessment and self-report in the French EPIMAN study, Akiskal et al. [31] proposed a redefinition of mania, in which four mood alterations were specified: elation, depression, anxiety, and irritability. Likewise, in a series of studies using different clinical assessment instruments, Henry and colleagues proposed that those in both manic and mixed states evidence an increase in all emotions [32–34]. They suggested that it might be more appropriate to define the mood in these patients as a function of intensity rather than as a function of tonality. In a subsequent study, the greatest emotional reactivity was demonstrated in manic and mixed patients compared to controls through an emotional induction protocol [35]. The fact that elevated mood, irritability, and depression–anxiety were distributed across different dimensions in our review suggests that mood in mania might depend on an orthogonal combination of these factors. Therefore, our results support the hypothesis that the fundamental mood experience in mania is a state of increased mood reactivity, which can manifest heterogeneously depending on the co-occurrence of different euphoric–dysphoric emotions of varying intensity.

Finally, a commonly identified factor in our assessed studies was one constituted by psychosis, poor judgment, and lack of insight. This finding could provide some support for the current specifier of mania with psychotic features in DSM-5. Altered sleep tended to appear as an isolated domain, while distractibility–another current B criterion–was evaluated in only a few studies.

Overall, based on the factor distribution and loadings of the variables included in our review, we propose a redefinition of the diagnostic criteria for mania to be evaluated in future studies (Table 3). It is noteworthy that, in line with some previous proposals of other authors [4,31], these criteria would allow the diagnosis of mania in the absence of an elevated or irritable mood.

**Table 3.** Tentative redefinition of the diagnostic criteria for DSM-5 mania to be evaluated in future studies (in parentheses the clinical features as defined in our review)

A distinct period of abnormality lasting at least 1 week (or any duration if hospitalization is necessary) in which five or more of the following criteria are met: A‒Increased activity, speech, and thought (at least one). ‒Increase in goal directed activity or psychomotor agitation (hyperactivity/increased sociality/increased sexuality/psychomotor agitation). ‒More talkative than usual or pressure to keep talking (increased speech). ‒Flight of ideas or subjective experience that thoughts are racing (thought disorder). B‒Increased mood reactivity (at least one). ‒Elevated or expansive mood (elevated mood). ‒Irritability (irritability). ‒Depressive mood (depressed mood). C‒Other manic features ‒Expansiveness, over-confidence, or grandiosity (grandiosity). ‒Decreased need for sleep (altered sleep). ‒Distractibility (distractibility). ‒Excessive involvement in activities that have a high potential for painful consequences (poor judgment). ‒Additional features (lack of insight/disruptive-aggressive behavior).

### Relationship between mania and mixed states

Mixed states are viewed as highly heterogeneous entities, and with the prevalence among manic patients varying from 14 to 67% [36]. This diversity is generally explained as reflecting a dimensional approach [33] or the existence of several categories of mixed states [37]. However, similar to mania, both the definition of mixed episodes in DSM-IV (1994), as well as the replacement of a mixed-state specifier in DSM-5 (2013), has been criticized for their lack of empirical support and of clinical consensus [38–40].

The two studies with larger sample sizes evaluated in our review had, in addition to patients with mania, patients diagnosed as having mixed episodes [9,12]. The main characteristics and the factors found in these mixed episodes samples are shown in Table 4. In both studies, the factorial structure in those with either manic or mixed episodes did not show a substantial difference. Moreover, the study by Swan et al. [12] was the only one that included a sample of patients with mixed episodes that were evaluated with an extensive list of manic and depressive items. The factors that emerged in that study were very similar to those found in our review of studies of mania (i.e., depressive-anxious, manic core features, irritability-aggressive behavior, and altered sleep). Therefore, the few empirical data available to date are compatible with the notion that manic and mixed states might not be different types of episodes, supporting the removal of the mixed episode and its replacement by the mixed specifier in DSM-5. Alternatively, it could be proposed that the fundamental difference could be a dimensional variation of mood, in which core features factor identified in our review (and therefore the elevated mood) predominates in “pure” mania, while depressive and irritable factors (and therefore depressive or irritable mood) dominate in “mixed” mania. This view agrees with the results of most studies that, using cluster analysis, have identified forms of pure (or classical or euphoric), dysphoric (or irritable or aggressive), and depressive mania [11–14,16]. The redefinition of the diagnostic criteria proposed in Table 3, as there were no restrictions due to the tonality of mood abnormalities would allow us to include within the category of mania all these dimensional variations. Comparison of the factorial structure of mania with and without mixed-state specifier could be the focus of future studies in order to further clarify this issue.

**Table 4.** Summary of studies in mixed states

Study	*n*/setting/diagnosis	Items/no. of factors	Rotation	Factor 1/variance	Factor 2/variance	Factor 3/variance	Factor 4/variance	Factor 5/variance	Total variance
Mixed studies									
[9]	71 mixed/int /DSM-IV	12 items Young Mania Rating Scale and total score from Hamilton Depression Rating Scale score/5	Varimax	Hyperactivity, increased speech/18%	Poor judgment, irritability/16%	Psychosis, elevated mood, lack of insight/15%	Thought disorder, depressive symptoms (HDRS score)/10%	Altered sleep/10%	69%
[12]	644 mixed/NS/DSM-IV	21 items Young Mania Rating Scale and Montogomey-Asberg Depression Rating Scale/5	Varimax	Non-reactivity mood, depressive mood, depressive feelings, fatigue, suicide, distractibility, appetite; psychomotor agitation/18.5%	Thought disorder, elevated mood, increased speech, psychosis/10.6%	Altered sleep/7.2%	Appearance, Lack of insight, increased sexuality/5.4%	Disruptive–aggressive behavior, irritability/6.7%	48.4%

The clinical features within each factor in the different studies were extracted in decreasing order of loadings (higher loading first). The clinical items from each individual study were undergoing to the harmonization procedure described for mania.

Abbreviations: Int, intpatients; NS, not specified; Out, outpatients.

Of course, the similarity in factorial structure across those with pure mania and those with mixed mania does not preclude the evidence that those with the former profile (so-called mixed states) are more difficult to treat, have a more severe course, and have a higher risk of suicide [41,42].

### Relationship between mania and depressive states

The current conceptualization of the positions of BDs hypo/mania and depression are at opposite poles of a unitary disease entity. Our findings on the structure of mania contradict that conception, since depression seems to be a relevant dimension that can form part of a manic state. In fact, it is important to note that the depressive dimension identified in some of our studies was seemingly not necessarily restricted to a mood abnormality such as “mood lability,” but included other manifestations of major depressive episode, such as depressive thoughts, fatigue, and anxiety or suicidality. The exact nature of the association between mania and depression remains speculative. Depressive symptoms could be inherent features of mania that manifest in a subset of bipolar patients. Alternatively, our findings might be compatible with the “two-illness model” of BD proposed by some authors [43,44], according to which major depression is a comorbid condition that can occur before, during, or after a manic episode (such as occurs in schizophrenia or others psychiatric/medical disorders). Moreover, two recent family studies [45,46] that demonstrated the independence of the inheritance pattern of mania and depression also challenged the traditional view of BD. We suspect that there are several contributing factors linking mania with depression. Future studies are needed to elucidate the nature of the depressive features found during manic episodes, and identify the key models.

Findings from the present study show that the core clinical features of mania appear at the opposite pole to key melancholic features rather than to those of the more heterogeneous major depressive episode construct. In a recent review, the clinical variables that best identified melancholic depression were mood non-reactivity, motor retardation, and retardation of speech and thought [47]. Therefore, mania and melancholia might be considered as opposite poles (increase and decrease, respectively) of three core features: mood-reactivity, motor activity, and speech and thought. This approach has points of agreement both with Kraepelin’s conception [48] and with recent proposals about mood disorder modeling [49,50]. Moreover, this highlights the need to address an under-studied topic in the BD field, such as differences in the clinical meaning (i.e., clinical course, family aggregation, and therapeutic response) of melancholic and non-melancholic depressions.

## Conclusions

Despite several limitations mentioned above, the current review summarizes the empirical evidence available on the factorial structure of mania. Results suggest a multidimensional structure of mania, in which hyperactivity, increased speech and thought disorder are key constructs. The abnormality of mood during mania could be heterogeneous and would depend on the co-occurrence of euphoric and dysphoric emotions.

The preliminary findings of our review may be a source of future research. Our results do not support the current primacy given to the elevated, expansive, or irritable mood in the definition of mania, so alternative definitions should continue to be empirically tested. Confirmatory factor analysis could be used to corroborate the general structure of pure and mixed mania. Studies should include relevant variables poorly studied to date, such as lability of mood, distractibility, and impulsivity. Our review was focused on mania. As most of the included studies were performed on inpatient populations, our results might not be representative of milder forms of mania present in outpatients. Moreover, although some authors argue that Bipolar I and Bipolar II disorders are the same illness [51,52], our preliminary results should not be extrapolated to the factor structure of hypomania, which could also be the focus of future studies. Clarification of the structural nature of mania would also be useful to evaluate the response to different treatments and to better understand the pathophysiological substrate of this disorder.

## Data Availability

The data supporting the findings of this study are available from the corresponding author (D.J.M.) on request.
